# Chitin-Based Porous Carbon Containing Cuprous Sulfide for Supercapacitor Electrode Materials

**DOI:** 10.3390/polym17233186

**Published:** 2025-11-29

**Authors:** Jiangyang Han, Wenchao Yu, Fukun Niu, Yang Hu, Hongmei Qin, Zhuqun Shi, Chuanxi Xiong, Quanling Yang

**Affiliations:** 1 Sanya Science and Education Innovation Park, Wuhan University of Technology, Sanya 572024, Chinahmeiqin@whut.edu.cn (H.Q.); zqshi2016@whut.edu.cn (Z.S.); xcx@whut.edu.cn (C.X.); 2 State Key Laboratory of Advanced Glass Materials, School of Materials Science and Engineering, Wuhan University of Technology, Wuhan 430070, China; 3 School of Chemistry, Chemical Engineering and Life Sciences, Wuhan University of Technology, Wuhan 430070, China; 4 Shenzhen Institute, Wuhan University of Technology, Shenzhen 518000, China

**Keywords:** chitin, cuprous sulfide, aerogel, supercapacitor

## Abstract

Chitin-derived biomass carbon materials are promising supercapacitor electrode materials due to their wide availability, low cost, high specific surface area, and nitrogen doping capability. However, their practical application is limited by insufficient conductivity and cyclic stability, often requiring functional modification or integration with complementary materials. In this study, we present a novel strategy by incorporating copper sulfide (Cu_2_S) into a chitin-based carbon matrix. Cu_2_S, known for its high intrinsic conductivity, excellent electroactivity, and theoretical specific capacity (~335 mAh·g^−1^), was uniformly doped into the three-dimensional carbon aerogel framework derived from chitin nanofibers (ChNF) through sol–gel, freeze-drying, and high-temperature carbonization processes. The resulting chitin-based carbon/Cu_2_S composite aerogel (CChNF/Cu_2_S) exhibited a hierarchical porous structure with Cu_2_S nanoparticles (20–30 nm) uniformly distributed on the carbon fiber surface. Electrochemical tests demonstrated its excellent performance, achieving a specific capacitance of 852 F·g^−1^ at 1 A·g^−1^, highlighting the synergistic effects of the conductive Cu_2_S and nitrogen-doped carbon framework for high-performance supercapacitor applications.

## 1. Introduction

Supercapacitors, with their ultra-high power density, exceptionally long cycle life, rapid charge and discharge capabilities, and wide operating temperature range, are regarded as up-and-coming next-generation energy storage devices [1,2,3]. Currently, these devices have been practically applied in various fields, including the energy recovery systems for rail transportation and electric vehicles, auxiliary power supplies, and support for low-temperature start-up [4,5,6]. Electrode materials are one of the key factors determining the electrochemical performance of supercapacitors. Compared to metal oxides (such as RuO_2_, which are costly) and conductive polymers (such as PEDOT, which have poor cycling stability), carbon-based materials have garnered significant attention due to their high specific surface area, excellent electrical conductivity, and good chemical stability, as well as abundant raw material sources and environmental friendliness [7,8,9,10,11,12,13]. These features make carbon-based materials promising for enhancing energy density while controlling costs, providing significant direction for the development of next-generation high-performance energy storage materials [14]. The raw materials for carbon-based materials are diverse, including gaseous carbon sources such as methane and carbon dioxide, waste plastics, various types of biomass, and natural graphite. Currently, in the face of the increasing depletion of fossil resources, biomass materials are receiving more attention in the field of carbon materials due to their renewability and environmental friendliness, which helps alleviate resource scarcity pressure and responds to the national “dual carbon” strategic goals [15,16].

Chitin, a natural polymer second only to cellulose, contains nitrogen (N) and oxygen (O) heteroatoms in addition to carbon and hydrogen [17]. These heteroatoms play a crucial role in reducing the band gap of carbon materials derived from chitin, enhancing their electrical conductivity and charge storage capacity. During the carbonization process, the volatilization of nitrogen and oxygen contributes to the formation of a hierarchical porous/mesoporous structure, significantly increasing the specific surface area and providing more pathways for ion storage and transport, which is highly beneficial for supercapacitor applications. While nitrogen doping improves the conductivity of chitin-derived materials, it is insufficient to fully address the inherent low conductivity issue. To overcome this limitation, researchers often incorporate other high-conductivity materials, such as polyaniline, carbon nanotubes, or metal oxides, into the composite. These additives enhance the overall conductivity and performance of the material, creating a synergistic network that improves electron mobility and addresses the low conductivity defect, ultimately offering a promising pathway for high-performance supercapacitor electrode materials [18].

In the past few decades, transition metal compounds have been regarded as ideal electrode materials for supercapacitors due to their ability to achieve charge storage through Faradaic redox reactions of transition metal cations and their high theoretical energy density. However, such materials often encounter issues such as slow electrochemical reaction kinetics, poor intrinsic electronic conductivity, insufficient cycling stability, and inadequate rate performance, which severely limit their practical applications [19,20,21,22,23]. Compared to transition metal oxides, sulfides (such as NiCo_2_S_4_ and α-NiS) typically exhibit higher conductivity and electrochemical activity, with some even displaying metallic-like conductive properties [24,25]. These characteristics significantly contribute to enhancing the rate performance and cycling stability of the materials. Based on this, to improve the relatively poor electrochemical performance of cuprous oxide (Cu_2_O), this study considers sulfide treatment, successfully producing cuprous sulfide (Cu_2_S). Cu_2_S belongs to the hexagonal crystal system and is a p-type semiconductor with a band gap of approximately 1.2 eV. This compound is composed of Cu^+^ and S^2−^ ions, with a theoretical capacity that is relatively high (approximately 335 mAh·g^−1^) [26]. Due to its high theoretical specific capacity, Cu_2_S is widely applied in the field of lithium-ion/sodium-ion battery electrode materials, as well as in solar cells and catalysis. However, Cu_2_S still faces several urgent issues as an electrode material: firstly, its inherent conductivity is insufficient, necessitating a composite with a highly conductive substrate to enhance overall conductivity; secondly, it has poor cycling stability [27,28]. These deficiencies make it difficult to utilize Cu_2_S alone as a high-performance supercapacitor electrode material.

Therefore, we prepared chitin-based carbon aerogels with excellent electrical conductivity, specifically chitin carbon aerogels/sodium copper sulfide (CChNF/Cu_2_S), by introducing copper sulfide (Cu_2_S) into chitin gel and conducting high-temperature carbonization. In this process, Cu_2_S coordinated with the amino (-NH_2_) groups on the surface of the chitin carbon aerogel, forming a hydrogel. Subsequently, a series of processes, including solvent replacement, freeze-drying, high-temperature carbonization, and high-temperature sulfidation, were employed to achieve the target product, the CChNF/Cu_2_S electrode material. The specific capacitance of this material reached 852 F·g^−1^, representing a 154% increase compared to pure CChNF’s 334.9 F·g^−1^. This achievement provides new possibilities for breakthroughs in the electrical conductivity of chitin-based electrode materials and holds significant importance for the innovative applications of biomass materials in the energy field.

## 2. Materials and Methods

### 2.1. Materials

Chitin (Zhejiang Golden Shell Pharmaceutical Co., Ltd., Yuhuan, China), sodium borohydride (≥98%, Aladdin Biochemical Technology Co., Ltd., Shanghai, China), sodium hydroxide (AR, Sinopharm Chemical Reagent Co., Ltd., Shanghai, China), hydrochloric acid (AR Sinopharm Chemical Reagent Co., Ltd.), sodium chlorite (≥80%, Aladdin Biochemical Technology Co., Ltd.), glacial acetic acid (GR, Sinopharm Chemical Reagent Co., Ltd.), copper acetate monohydrate (AR, 99%, Aladdin Biochemical Technology Co., Ltd.), Copper acetate monohydrate (AR, 99%, Aladdin Biochemical Technology Co., Ltd.), anhydrous ethanol (AR, Tianjin Fuyu Fine Chemical Co., Ltd., Tianjin, China), tert-butanol (AR, Aladdin Biochemical Technology Co., Ltd.), sublimated sulfur (AR, ≥99.5%, Aladdin Biochemical Technology Co., Ltd.), *N*,*N*-dimethylformamide (>99.9%, Aladdin Biochemical Technology Co., Ltd.), Nafion solution (≥ 99.9%, Aladdin Biochemical Technology Co. Ltd.), Nafion solution (5 wt.%, Kunshan Yier Sheng International Trading Co., Ltd., Kunshan, China), γ-alumina powder (0.05 μm, Shanghai Chenhua Instrument Co., Ltd., Shanghai, China), white nylon polishing cloth (Shanghai Chenhua Instrument Co., Ltd.).

### 2.2. Purification of Chitin

Twenty grams of chitosan powder are weighed and added to a 1 mol·L^−1^ NaOH solution. The mixture is stirred at room temperature for 24 h, after which it is subjected to vacuum filtration. The resulting chitosan is transferred to a 0.5 mol·L^−1^ HCl solution and stirred at room temperature for an additional 24 h to remove inorganic salt impurities. The chitosan is then rinsed with deionized water until the washings are neutral (pH ≈ 7) [29,30]. The wet chitosan is placed in 1000 mL of a 0.3% sodium chlorite solution adjusted to pH 3.5, and the suspension is stirred in an 80 °C water bath for 5–6 h to achieve decolorization; a yellow-green color change is observed. After the decolorization step, the chitosan is rinsed again with deionized water until the washings are neutral [31,32]. Finally, freeze-dry the wet chitosan for 48 h to obtain purified chitosan powder.

### 2.3. Preparation of Chitin Nanofiber Dispersion

1.1 g of purified chitin powder is weighed, and 30 mL of a 33% NaOH aqueous solution, together with 0.03 g of NaBH_4_, is added. The mixture is stirred at 90 °C for 3 h. After the reaction is completed, the suspension is centrifuged at 8000 rpm for 10 min. The resulting precipitate is redispersed in an appropriate volume of deionized water, and the centrifugation-washing cycle is repeated 3–5 times until the wash solution reaches neutrality (pH ≈ 7). The washed dispersion is then titrated with dilute acetic acid to pH 3.5, and deionized water is added to bring the total volume to 1000 mL, producing a translucent dispersion. Finally, this dispersion is homogenized using a high-pressure homogenizer, yielding a transparent ChNF dispersion [31,33].

### 2.4. Preparation of CChNF/Cu_2_S Carbon Aerogels

**Figure 1:**
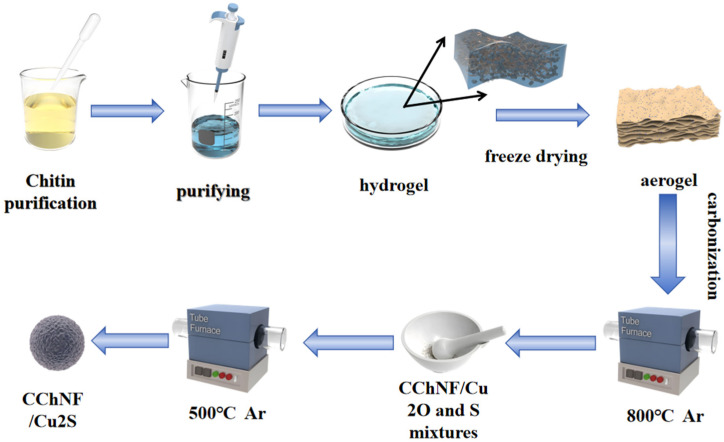
Schematic of the synthesis steps of CChNF/Cu_2_S preparation.

The alcogel is pre-frozen at −80 °C for 12 h and subsequently freeze-dried for 24 h. It is thereafter heated in an argon flow (50 sccm) at a temperature ramp of 5 °C min^−1^ to 300 °C and held for 1 h for pre-oxidation, followed by carbonization at 800 °C for 2 h, yielding CChNF/Cu_2_O (the control sample prepared without copper salt yields CChNF) [34,35,36]. CChNF/Cu_2_O is ground together with sublimated sulfur in a mass ratio of 1:1, and the mixture is heated under argon at a rate of 5 °C min^−1^ to 500 °C for sulfuration for 2 h. Afterwards, the material is cooled to 300 °C and held for 6 h to remove residual sulfur [37,38]. The products are labeled according to the initial mole ratio as CChNF/Cu_2_S-1 (1:1) and CChNF/Cu_2_S-2 (1:2) (Figure 1).

### 2.5. Characterization

The internal micro-morphology of carbon aerogels was observed using a Zeiss Ultra Plus field emission scanning electron microscope (Zeiss GmbH, Oberkochen, Germany). The internal micro-morphology and lattice fringe of carbon gels were examined using a JEM-2100F field emission high-resolution transmission electron microscope (Japan Electronics Corporation, Tokyo, Japan). A D8 Advance X-ray diffractometer (BRUCK AXS Company, Karlsruhe, Germany ) was employed for phase analysis of carbon aerogels.

Before electrochemical performance testing, the surface of the glassy carbon electrode was carefully polished with alumina powder to remove any impurities. Subsequently, the sample powder was dispersed in DMF, and Nafion solution was added as a binder. Ultrasound treatment was performed for 30 min to achieve uniform dispersion. The prepared dispersion was then dropped onto the electrode surface, and after the solvent evaporated, the electrode was ready for performance characterization. Performance of the electrode materials was characterized using a CHI 660E electrochemical workstation (Shanghai Chenhua Instrument Co., Ltd., Shanghai, China) in a three-electrode system, with the working electrode being the test electrode, Hg/HgO (1 M KOH) as the reference electrode, and platinum wire as the counter electrode, with a 1 M KOH aqueous solution as the electrolyte. Techniques used include electrochemical impedance spectroscopy (EIS, frequency range 0.01 Hz to 100 kHz), cyclic voltammetry (CV, scan rate 1–100 mV·s^−1^), and galvanostatic charge–discharge (GCD, current density 1–20 A·g^−1^).

## 3. Results

### 3.1. Structural and Morphological Characterization

**Figure 2:**
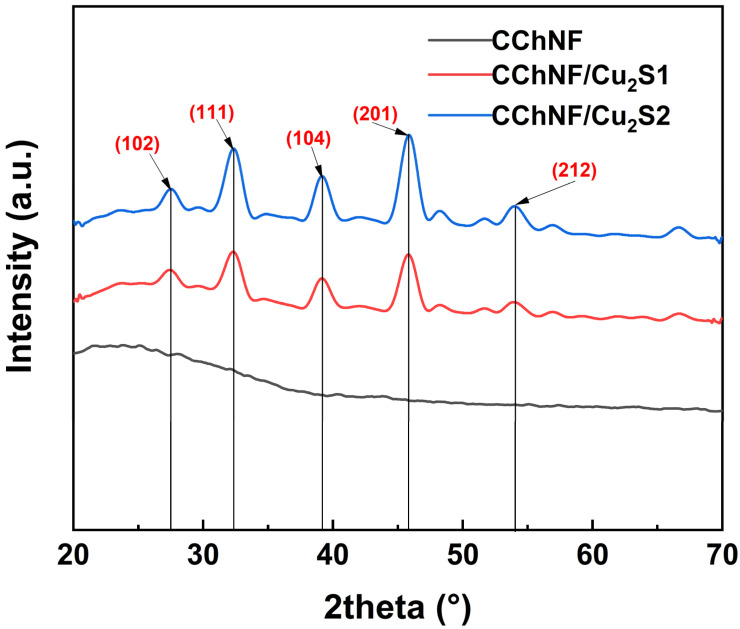
XRD spectra of different compositions of CChNF/Cu_2_S1, 2 and CChNF.

As shown in the XRD patterns (Figure 2), the introduction of Cu_2_S in the CChNF/Cu_2_S-1 and CChNF/Cu_2_S-2 composites results in identical characteristic diffraction peaks, with highly similar peak positions and intensities. Distinctive diffraction peaks corresponding to the (102), (111), (104), (201), and (212) planes of Cu_2_S were observed at 2θ = 27.5°, 32.3°, 39.1°, 45.8°, and 54.0°, respectively, clearly indicating the presence of the Cu_2_S phase. No characteristic peaks of elemental sulfur were observed, confirming the complete removal of residual sulfur. Furthermore, no impurity peaks of CuO or CuS were detected, which verifies that Cu_2_O and residual CuO were fully converted into Cu_2_S during the sulfidation process. This transformation is attributed to the decomposition of the intermediate product CuS, formed from CuO, at 220 °C into /Cu_2_S and elemental sulfur. For the CChNF carbon aerogel, a broad and weak peak around 2θ ≈ 43–44° corresponds to the (100) plane of graphite, indicating an amorphous carbon structure with a high density of planar defects. The lattice distortions caused by the incorporation of heteroatoms, such as nitrogen, further broaden the diffraction peaks and introduce shoulder peaks. These structural defects provide active sites for pseudocapacitive reactions.

**Figure 3:**
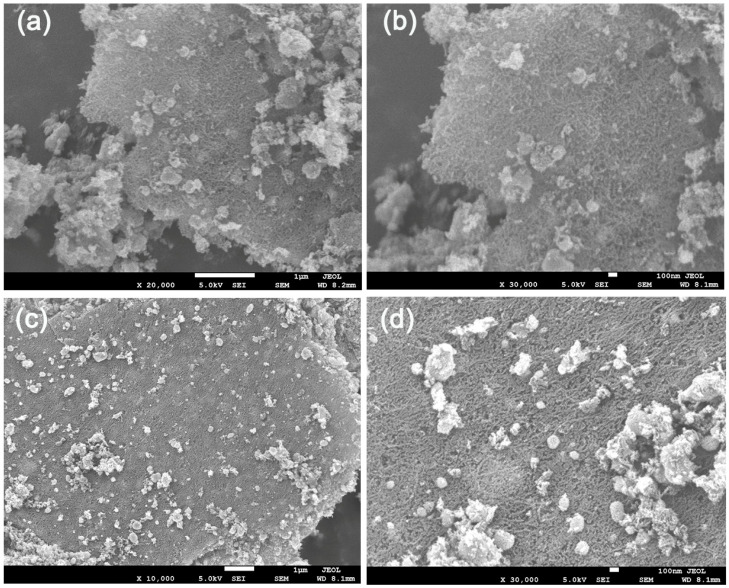
SEM images of (**a**,**b**) CChNF/Cu_2_S-1 and (**c**,**d**) CChNF/Cu_2_S-2.

Figure 3 displays the SEM morphology of CChNF/Cu_2_S carbon aerogels with different components. The observations indicate that the distribution of Cu_2_S within the carbon aerogel matrix is non-uniform, resulting in the formation of irregular ‘spherical’ structures, accompanied by a certain degree of agglomeration. At the same time, the crystal size distribution of Cu_2_S is relatively broad, with diameters ranging from several tens of nanometers to several hundred nanometers. Compared to the uniformly dispersed Cu_x_O/Cu_2_O precursors before sulfuration, the Cu_2_S produced by the sulfuration reaction shows a significant decrease in both spatial distribution and particle size uniformity. This morphological characteristic may have detrimental effects on the rate of performance and cycling stability of the material. The cause is hypothesized to be related to the sublimation of sulfur undergoing a liquid phase during the secondary heating process: liquid sulfur fills the pores of the carbon aerogel, leading to the contact and reaction of the originally well-dispersed Cu_x_O/Cu_2_O particles. However, it is worth noting that a considerable portion of the Cu_2_S crystals are only a few tens of nanometers in size. As a typical battery-type material, reducing the size of Cu_2_S to the nanoscale can induce it to exhibit pseudocapacitance behavior, characterized by rapid surface redox kinetics. Therefore, although the microstructure of the CChNF/Cu_2_S carbon aerogel may not have reached an ideal state, its electrochemical performance results may not necessarily be restricted by this.

**Figure 4:**
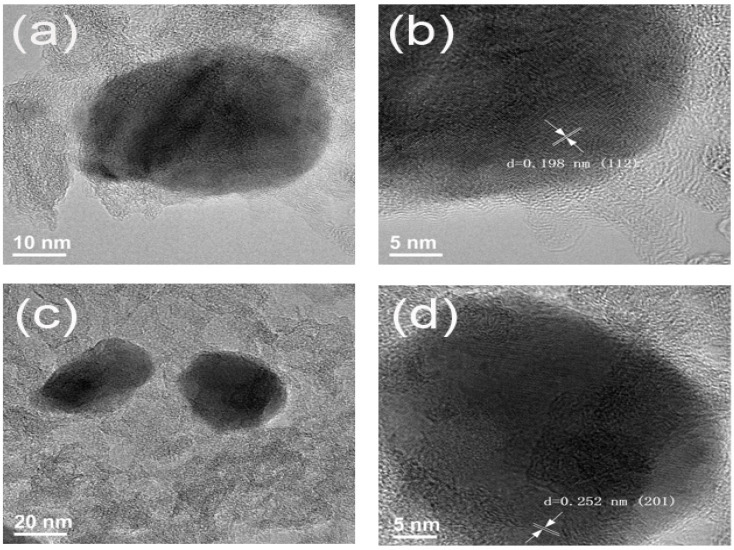
HRTEM images of (**a**,**b**) CChNF/Cu_2_S-1 and (**c**,**d**) CChNF/Cu_2_S-2.

To obtain more detailed microstructural information of the materials, high-resolution transmission electron microscopy (HRTEM) was conducted on the CChNF/Cu_2_S carbon aerogel (Figure 4). Given the wide distribution of the crystal size in Cu_2_S, representative crystals were selected for observation.

As shown in Figure 4a,b, the Cu_2_S crystals exhibit an ellipsoidal structure with a diameter of approximately 20–30 nm. Lattice fringe analysis was performed using high-resolution transmission electron microscopy (HRTEM). As indicated by the arrows, the measured interplanar spacing is d = 0.198 nm, corresponding to the (112) crystal plane of Cu_2_S, which is consistent with the XRD characterization results. Correspondingly, Figure 4c,d presents typical regions of the CChNF/Cu_2_S-2 sample, where Cu_2_S crystals predominantly maintain an ellipsoidal morphology. The lattice fringe measurement results in this region indicate d = 0.252 nm, which corresponds to the (200) crystal plane of Cu_2_S.

### 3.2. Electrochemical Performance Evaluation

**Figure 5:**
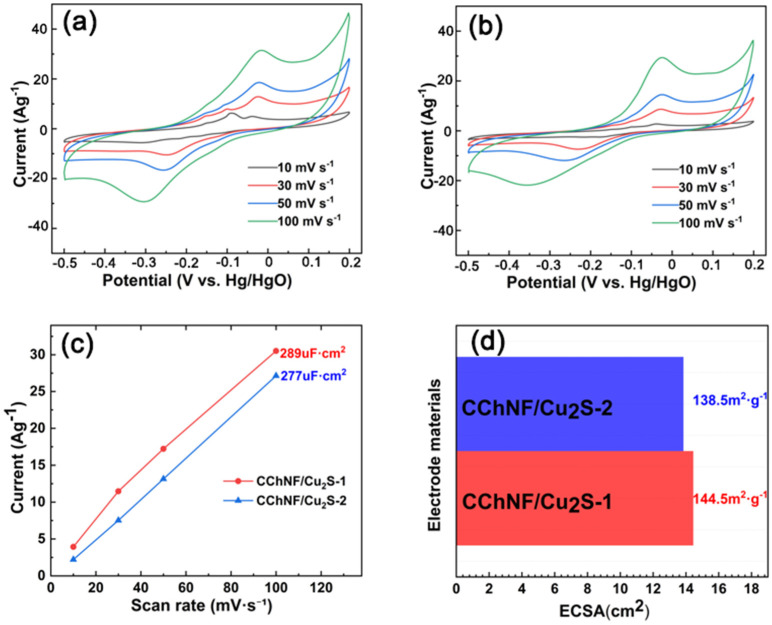
CV curves of (**a**) CChNF/Cu_2_S-1, (**b**) CChNF/Cu_2_S-2 at various scan rates, (**c**) the Cdl values of the electrodes obtained from the CV curves in the non-Faraday region, and (**d**) comparison of the ECSAs of the electrodes.

Cyclic Voltammetry (CV) is a widely used electrochemical characterization technique, the principle of which involves applying a cyclic scanning voltage to the working electrode and recording the relationship between the response current and the scanning voltage [39]. The CV curve can reveal the voltage window of the electrode material, the reversibility of the reaction, and the characteristic redox peaks of pseudocapacitive materials [40]. Based on this, CV tests were conducted on CChNF/Cu_2_S carbon aerogels with different components within the scan rate range of 10 to 100 mV/s (Figure 5). As shown in Figure 5a,b, all samples exhibit significant redox peaks, which can be attributed to the redox reaction between Cu_2_S and OH^−^ ions in the electrolyte. The redox peaks demonstrate good symmetry, indicating that Cu_2_S possesses excellent reaction reversibility. Further analysis of the CV curves at low scanning speeds reveals the presence of multiple smaller redox peaks in the CChNF/Cu_2_S samples. This phenomenon may stem from the multi-step redox reaction process of Cu_2_S, which is akin to the stepwise conversion reactions of polysulfides in lithium-sulfur batteries, and is generally advantageous for enhancing the specific capacity of the material [41]. Furthermore, the broad size distribution of Cu_2_S crystals leads to differences in the reaction kinetic rates of particles with varying diameters, which may also be the reason for the characteristics of stepwise reactions.

The electrochemically active surface area (ECSA) is another important parameter influencing the electrode’s electrochemical performance, which can be calculated from the electric double layer capacitance (Cdl) according to Equation (1). Figure 5c displays the Cdl values of the electrodes obtained from the CV curves in the non-Faraday region (Figure 5a,b), and the corresponding ECSAs are compared in Figure 5d. The ECSA of CChNF/Cu_2_S-1 and CChNF/Cu_2_S-2 is 144.5 m^2^·g^−1^ and 138.5 m^2^·g^−1^, respectively. It can be seen that the CChNF/Cu_2_S-1 electrode material has a higher utilization rate, which is because part of the copper sulfide is wrapped by carbon nanofibers and embedded within the material, partially blocking the internal pores. This also corresponds to the previous SEM observations. The addition of cuprous sulfide is not necessarily better in larger amounts.

(1) $$ECSA=\frac{Cdl}{K}\;$$

In the formula, *ECSA* refers to the electrochemically active surface area (m^2^·g^−1^), *Cdl* is the double-layer capacitance (µF·cm^2^), and *K* is the proportionality constant (mF·cm^−2^). In this study, 1 M KOH was used as the electrolyte, so the *K* value was taken as 0.020 mF·cm^−2^, which is widely used in the literature.

Galvanostatic Charge–Discharge (GCD) is another conventional electrochemical characterization technique. This method applies a constant current to the working electrode and records the variation in its potential over time [42]. In the evaluation of electrode performance, the current density applied (i.e., mass-specific current) is typically calculated based on the mass of the active material, which is a key parameter. For supercapacitors that are predominantly governed by double-layer capacitive behavior, the energy storage mechanism dictates that the GCD curve displays a symmetrical triangular shape. This is because, under constant current conditions, the electrode potential changes linearly over time. However, for electrode materials that exhibit significant pseudocapacitance behavior, corresponding charge/discharge platforms will appear on the GCD curve due to the involvement of redox reactions.

When the discharge process exceeds approximately 1500 s, the battery voltage experiences a sudden and significant drop. This phenomenon is mainly due to self-discharge, parasitic Faradaic reactions, and the gradual increase in the equivalent series resistance (RESR) caused by incomplete wetting of the hierarchical porous carbon structure. 1. Around the 1500 s, the electrode potential reaches a critical point at which functional groups such as quinone and amino groups on the surface of chitosan-derived carbon aerogels undergo irreversible electrochemical reduction reactions. 2. After approximately 1500 s of continuous charging and discharging, ion transport in the micropores of the carbon material encounters a bottleneck. Meanwhile, volume changes during the charging and discharging process may increase the internal contact resistance within the electrode. These factors collectively cause a sharp increase in the internal resistance (Rs) of the electrode, leading to a transient non-capacitive current spike and a sudden drop in potential on the GCD curve.

Moreover, the symmetry of the GCD curve depends on the reversibility of the material’s redox reactions, and thus it is not always symmetrical [41,42]. Typically, the specific capacitance of the electrode material can be calculated from the GCD curve using the following formula:

(2) $$C=\frac{I\Delta t}{m\Delta V\;}$$

**Figure 6:**
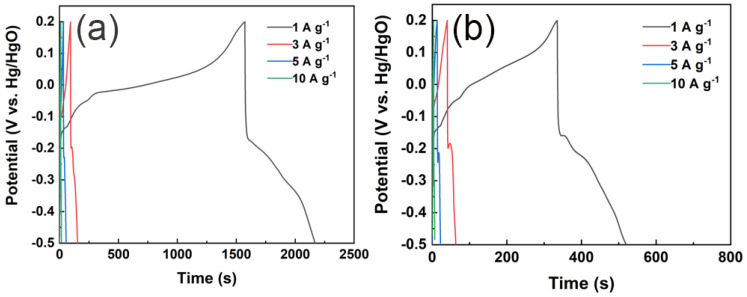
GCD curves of (**a**) CChNF/Cu_2_S-1 and (**b**) CChNF/Cu_2_S-2 at different current densities.

Among them, (*C*) represents the specific capacitance (F·g^−1^), (I) denotes the discharge current (A), (Δ*t*) indicates the discharge time (s), (m) stands for the mass of the active material (g), and (Δ*V*) refers to the voltage window during the discharge process (*V*). Based on this, GCD tests were conducted on CChNF/Cu_2_S carbon aerogels with different current densities (Figure 6). As shown in Figure 6a,b, all sample GCD curves display significant charge and discharge platforms. Notably, at a current density of 1 A·g^−1^, multiple charge and discharge platforms can be observed, which directly confirms the multi-step oxidation-reduction reaction process of Cu_2_S as inferred from the CV tests. By comparing the discharge times at a current density of 1 A·g^−1^, it is found that CChNF/Cu_2_S-1 has the longest discharge time, indicating that it possesses the highest specific capacitance. Although CChNF/Cu_2_S-2 contains a higher proportion of Cu_2_S, its severe stacking behavior significantly limits the effective utilization of capacitance. According to calculations, the specific capacitance of CChNF/Cu_2_S-1 reaches 852 F·g^−1^ at 1 A·g^−1^, significantly higher than that of CChNF/Cu_2_S-2, which is 264 F·g^−1^, and even lower than that of pure CChNF, which is 334.9 F·g^−1^. This anomalous phenomenon can be attributed to two factors: (1) the stacking of Cu_2_S particles hinders the effective contact of electrolyte ions and charge transfer. (2) The high density of Cu_2_S results in a relatively reduced content of conductive carbon nanofibers in CChNF/Cu_2_S-2 for the same mass, which further decreases its overall capacity contribution [26].

In addition, the performance analysis of the magnification shows that as the current density increases to 3 A·g^−1^, the specific capacitance of CChNF/Cu_2_S-1 decreases to 258.9 F·g^−1^ (with a capacity retention rate of 30.4%), indicating a significant drop. This primarily stems from the complexity of the internal Cu_2_S crystal size and distribution: on one hand, the presence of large-sized Cu_2_S crystals and their stacking effects restricts ion diffusion and reaction kinetics [24]. This synergistic effect ultimately determines the capacity retention characteristics of the material under high current density.

**Figure 7:**
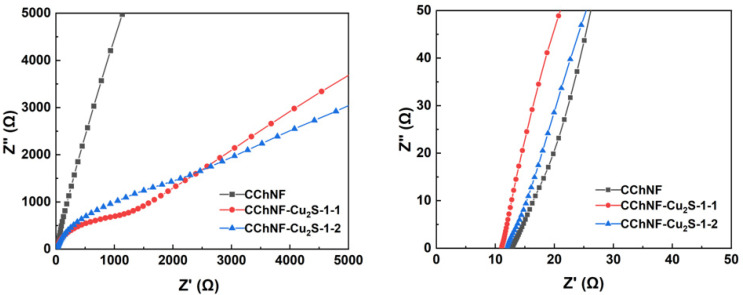
EIS spectra of different compositions of CChNF/Cu_2_S and CChNF.

Figure 7 shows the Nyquist plots of CChNF/Cu_2_S carbon aerogels with different compositions and pure CChNF at open-circuit potential over a frequency range of 0.01 Hz to 100,000 Hz. All samples have a real-axis intercept of approximately 12 in the high-frequency region, indicating that this series of chitosan-derived Cu_2_S/carbon aerogel composites possesses relatively low equivalent series resistance (ESR). Notably, CChNF/Cu_2_S-1 and CChNF/Cu_2_S-2 exhibit similar Faradaic impedance, as inferred from their impedance behavior at low frequencies. This phenomenon can be attributed to the similar microstructures revealed by SEM and HRTEM characterization (ellipsoidal particles, wide size distribution, and degree of stacking), which do not lead to significant changes in Warburg impedance due to differences in crystal structure. However, the relatively high resistance is also a result of the aggregation of Cu_2_S stacks and the inherently insufficient conductivity of the CChNF matrix, which remains an important direction for future research breakthroughs.

**Figure 8:**
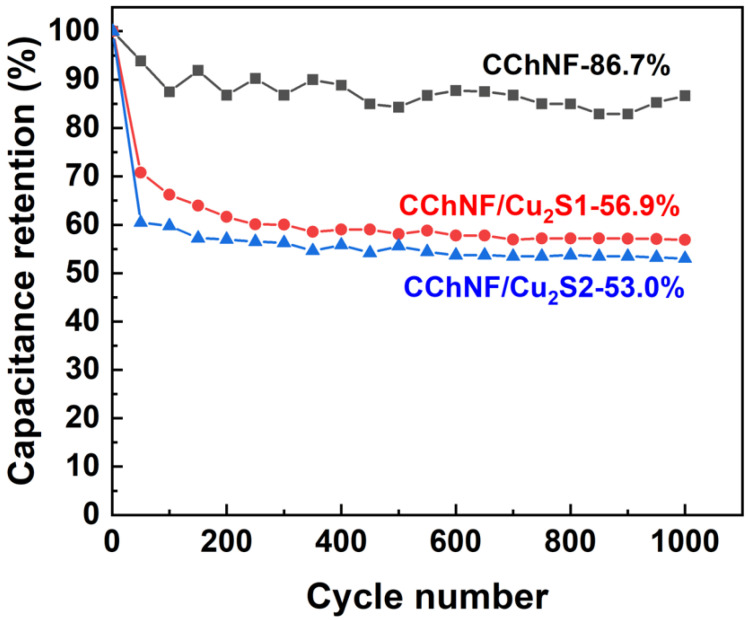
Cycling stability of different compositions of CChNF/Cu_2_S and CChNF at 3 A·g^−1^ over 1000 cycles.

Figure 8 shows the capacity retention rates of different components, CChNF/Cu_2_S carbon aerogels, and pure CChNF after 1000 cycles at a current density of 3 A·g^−1^. The capacity retention rates of CChNF/Cu_2_S-1 and CChNF/Cu_2_S-2 are only 56.9% and 53.0%, respectively. This significant capacity decay may stem from the inhomogeneity in the distribution and size of Cu_2_S crystals. Although Cu_2_S exhibits intrinsically superior electrical conductivity and reversible reactivity compared to Cu_2_O, the presence of large-sized crystals and their stacked structure severely restricts the full utilization of the active material and the transport of ions/electrons, hindering the effective release of capacitance [37]. Well-dispersed small-sized crystals can contribute more effectively to their charge storage capacity. This study indicates that future improvements in the cycling stability and overall electrochemical performance of the composite materials will require precise control over the size of Cu_2_S crystals (particularly achieving uniformity at the nanoscale) and optimizing their dispersibility.

### 3.3. Investigation of Charge Storage Mechanism

Based on various characterizations and electrochemical analyses, the excellent capacitive performance of CChNF/Cu_2_S arises from the synergistic contribution of electric double-layer capacitance (EDLC), pseudocapacitance, and ion intercalation mechanisms. Among them, the EDLC is formed by the oxygen- and nitrogen-containing functional groups on the material’s surface interacting with electrolyte ions via hydrogen bonding and π-π interactions, creating a stable double layer. CV curves at different scan rates exhibit near-rectangular shapes, which is a typical feature of double-layer capacitance. The GCD curves display highly symmetric triangular shapes, further confirming its excellent reversibility and the dominance of double-layer charge storage. Pseudocapacitance (Faradaic) involves rapid, reversible Faradaic redox reactions occurring on or near the material’s surface. The presence of pronounced redox peaks in the high potential region of the CV curves and the capacitive arcs in the high-frequency region of the EIS spectra indicates a pseudocapacitive contribution. SEM images reveal a rich porous structure, where micropores greatly increase the specific surface area, mesopores serve as rapid ion transport channels, and macropores act as ion buffers. This architecture allows ions to enter and exit efficiently, thereby facilitating charge storage.

In summary, the charge storage mechanism of CChNF/Cu_2_S is a synergistic process. The three-dimensional conductive carbon framework ensures fast electron transport, providing a foundation for both double-layer capacitance and pseudocapacitive reactions. The double-layer mechanism contributes high power density and excellent cycling stability, while the pseudocapacitive mechanism significantly enhances the material’s specific capacitance and energy density. Ultimately, a charge storage mechanism dominated by EDLC and pseudocapacitance, supplemented by ion intercalation, is established.

## 4. Conclusions

In this study, chitin was used as a carbon matrix, and copper sulfide (Cu_2_S) was successfully incorporated through a series of processes, including freeze-drying, high-temperature calcination, and sulfurization. The resulting chitin/Cu_2_S composite aerogel demonstrated outstanding electrochemical performance, achieving a specific capacitance of 852 F·g^−1^ at a current density of 1 A·g^−1^, which significantly outperformed the 334.9 F·g^−1^ of pure chitin aerogel. The composite maintained excellent cycling stability. The superior performance of the material can be attributed to the synergistic effects of the chitin carbon aerogel’s high specific surface area and porous structure, as well as the enhanced specific capacitance of Cu_2_S. However, the composite exhibited irregular, aggregated “spherical” Cu_2_S particles with uneven distribution, particularly noticeable in the CChNF/Cu_2_S-2 sample, where the high loading of Cu_2_S resulted in a densely stacked structure that limited the utilization of active materials. While Cu_2_S’s excellent electrical conductivity and reversible reaction characteristics contributed to its electrochemical performance, the presence of large, aggregated crystals hindered the efficient release of capacitance. Future research will focus on optimizing dispersion strategies, such as achieving uniform nanostructuring, to suppress aggregation and further enhance the overall electrochemical performance of the composite material.

## Data Availability

The original contributions presented in this study are included in the article. Further inquiries can be directed to the corresponding author.
